# Wettability alteration of calcite oil wells: Influence of smart water ions

**DOI:** 10.1038/s41598-017-17547-z

**Published:** 2017-12-12

**Authors:** Sanjay Prabhakar, Roderick Melnik

**Affiliations:** 1 The MS2Discovery Interdisciplinary Research Institute, M2NeT Laboratory, Wilfrid Laurier University, Waterloo, ON N2L 3C5 Canada; 2 The Department of Physics and Astronomy, University of Nebraska, Lincoln, NE 68588 USA

## Abstract

Further enhancement of crude oil recovery in the enhanced recovery stage from calcite oil wells is a major global challenge for oil industry. Experimental results suggest that ions present in sea water, also called smart water, have a significant influence on the wettability alteration (less oil wet) of calcite surface. In this paper, by utilizing Density Functional Theory (DFT) and Quantum Molecular Dynamics (QMD) simulations, we investigate the effect of additive ions of sea water in oil recovery by using acetic acid as a model compound of crude oil molecules. We find that Na^+^ ions precipitate to the calcite surface and form Na acetate. The binding energy of Na acetate is larger than original oil molecule (acetic acid), which reduces oil recovery. On the other hand, Mg^2+^ and ${\rm{S}}{{\rm{O}}}_{4}^{2-}$ ions can also reach to the calcite surface in proximity and modify the calcite surface. The binding energy of oil molecule on modified calcite surface is smaller than on pure calcite surface, which enhances oil recovery. Our results might help in understanding interaction among oil, water and additives ions of smart water for further experimental investigations.

## Introduction

Enhancement of crude oil recovery from oil wells is one of the global major challenges for oil companies. The enhancement of crude oil recovery can be achieved by injecting natural gases, bio-minerals, tap water, sea water, high pH solutions of surfactants, low salinity water, microbial and thermal methods^1–13^. Typically, there are three possible steps that can be applied during recovery of crude oil. These stages are: primary recovery, secondary recovery and enhanced recovery. In the primary recovery stage, due to large underground pressure of the oil reservoir, the crude oil can be extracted simply by drilling all the way to the reservoir where the recovery rate is up to 15%. When the underground pressures get down after the primary recovery stage, secondary methods are applied by injecting water or natural gases (air and carbon dioxide) to increase the reservoir pressure, which enhances the oil recovery rate by up to 45%. Finally, in the enhanced recovery stage, high pH solutions of surfactants, low salinity sea water are injected that cause the enhancement of oil recovery by up to 60%. This enhancement is believed to occur by wettability alteration of rock surfaces towards more hydrophilic.

In calcite oil wells, the calcite surfaces are more oil-wet than water-wet due to the presence of long-chain hydrocarbons ending with a carboxyl (-COOH) group. The typical binding energy of such hydrocarbons is larger than water molecules that induce more oil wet calcite surface^4,14–18^. Hence, in the enhanced recovery stage, when sea water is injected in the oil wells, contains of active sea water ions Mg^2+^, Ca^2+^, and ${\rm{S}}{{\rm{O}}}_{4}^{2-}$ affect the characteristics of wettability alteration of calcite surface (hydrophobic to hydrophilic) that enhance the efficiency of crude oil recovery^2–4^. The research articles by Zhange *et al*.^2^ and Fathi *et al*.^3^ found that injecting sea water in oil wells enhance the crude oil recovery up to 60%. For example, Zhange *et al*. have shown that injecting Mg^2+^ alone in oil wells enhance oil recovery to 20%^2^. Since the temperature was kept constant when Mg^2+^ ion was injected in oil wells, the 20% oil recovery is caused by the activity of Mg^2+^ alone but not due to fluid expansion. Similarly, injecting Ca^2+^ ion along with ${\rm{S}}{{\rm{O}}}_{4}^{2-}$ ion enhances oil recovery up to 32%. The oil recovery is further enhanced up to 42% when Mg^2+^ ion along with ${\rm{S}}{{\rm{O}}}_{4}^{2-}$ ion is injected in oil wells. Similar enhancement of oil recovery due to pouring sea water in oil wells was observed by Fathi *et al*.^3^. Further, Fathi *et al*.^3^ showed that low salinity sea water (removing some of NaCl) also caused an enhancement of crude oil recovery. Sakuma *et al*. provided theoretical confirmations that additive ions of sea water act as a surface modifier of calcite (Mg^2+^ replaces Ca^2+^ and ${\rm{S}}{{\rm{O}}}_{4}^{2-}$ replaces ${\rm{C}}{{\rm{O}}}_{3}^{2-}$), where relative binding of acetic acid (binding energy difference between water and acetic acid) as a representation of oil molecule is decreased. This cause enhancement of crude oil recovery due to less oil wet calcite surface. Our current work is different than Sakuma *et al*.^4^ in that the concentrations of Mg^2+^ is larger than ${\rm{S}}{{\rm{O}}}_{4}^{2-}$ ions in sea water, we show that additional Mg^2+^ can reach to the modified calcite surface and form Mg acetate, which is less sticky on the modified calcite surface. Also, theoretical predictions of enhancement of crude oil recovery due to pouring low salinity water in oil wells is not reported before.

In this paper, we consider acetic acid as a model compound of crude-oil molecule^2,4^ and investigate the influence of additive ions, Ca^2+^, Mg^2+^, ${\rm{S}}{{\rm{O}}}_{4}^{2-}$ and Na^+^ in oil recovery. We use quantum mechanical calculations based on density functional theory (DFT) and Quantum Molecular Dynamics (QMD) simulations and find that the binding energy of acetic acid is larger than water molecule on pure calcite surface, which confirms that the calcite surface is oil wet. We further find that Na^+^ ion reach to the calcite surface as a precipitate and form Na acetate that has larger binding energy than acetic acid. This provides an explanation for the experimental observations by Fathi *et al*. that injecting low salinity water (removing some of Na^+^ and Cl^−^ ions from sea water) in oil wells enhances oil recovery. As discussed by authors in refs^2,4^, oil recovery is enhanced when Mg^2+^ ion of sea water replaces Ca^2+^ of calcite and ${\rm{S}}{{\rm{O}}}_{4}^{2-}$ ion of sea water replaces ${\rm{C}}{{\rm{O}}}_{3}^{2-}$ of calcite. In this case, on modified calcite.MgSO_4_ surface, the binding energy of acetic acid decreases and water molecule increases. Such interplay causes an enhancement of crude oil recovery on modified calcite.MgSO_4_ surface. In sea water, the concentration of Mg^2+^ ions is the largest, about twice the concentration of ${\rm{S}}{{\rm{O}}}_{4}^{2-}$ and about four times the concentration of Ca^2+^. There are also some left over Ca^2+^ ions when Mg^2+^ replaces Ca^2+^. Hence, in this paper we find an additional role of Mg^2+^ and Ca^2+^ ions on modified calcite.MgSO_4_ surface. Our study shows that Mg^2+^ ions can approach near the oil molecule and form Mg acetate on modified calcite.MgSO_4_ surface, that also cause enhancement of crude oil recovery.

## Computational Methods

Density Functional Theory (DFT) calculations and Quantum Molecular Dynamics (QMD) simulations are performed under periodic boundary conditions, which are implemented in the Quantum Espresso software package^19^. Ultrasoft pseudopotentials and plane wave basis set with a kinetic energy and charge density cut-off at 60 Ry and 600 Ry are used. We include exchange and correlation effects within Perdew-Burke-Ernzerhof (PBE) Functional^20^. Van der Waals interactions are also included with the Semiempirical Grimme’s DFT-D2 corrections term^21^. For *H*_2_ *O* and acetic acid molecule on calcite surface, we use a 3.97 Å × 5.02 Å × 30.75 Å supercell that contains three layers (60 atoms) of orthorhombic (2 × 2) calcite slab model. For Ca and Mg acetates, we use a 7.95 Å × 5.02 Å × 30.75 Å supercell that contains three layers (120 atoms) of orthorhombic (4 × 2) calcite slab model. During geometry optimization, all atoms except the bottom layer are fully relaxed until the forces on atoms are smaller than 0.01 eV/Å. We have tested several k-point sampling. Calculation at Γ-point sampling fulfills convergence criteria. The optimized lattice constant of calcite is 3.97 Å along x-direction and 5.02 Å along y-direction which are in good agreement to the experimental data^22^. Since initial configuration of a molecule is important for finding the global minimum energy configuration, we performed quantum molecular dynamics simulations to find a reasonable initial configuration of adsorbed molecules on calcite surface. To be sure that we eventually reached the overall minimum energy configuration, 21 possible initial configurations were tested and lower energies were selected to find biding energies of adsorbed molecule on calcite surface. The VESTA program was used to draw the molecular structure^23^. The binding energy is calculated as:

1 $${E}_{b}={E}_{surf}+{E}_{mol}-{E}_{surf\mathrm{.}mol},$$

where *E*_*sur f*.*mol*_ is the total energy of adsorbed molecule on calcite surface, *E*_*sur f*_ is the total energy of calcite surface alone and *E*_*mol*_ is the total energy of molecule in a vacuum. The total energies of free molecules are calculated in a large size of vacuum (20 Å × 20 Å × 20 Å) by using same k-point sampling at Γ-point. The distances between the molecule and the boundaries are kept more than 8 Å to avoid any artifact interaction between molecules. For Quantum Molecular Dynamics (QMD) simulatons, we used 57 *H*_2_ *O* molecules in a (7.94 Å × 10.04 Å × 21.23 Å) size of supercell, which corresponds to the experimental density of water, 1 kg/L. For QMD, we have used 0.96 femo-second as an each ionic time step, temperature re-scales to 350 K and used the same k-point sampling and energy cut-off that were used for DFT calculation.

## Results

**Figure 1:**
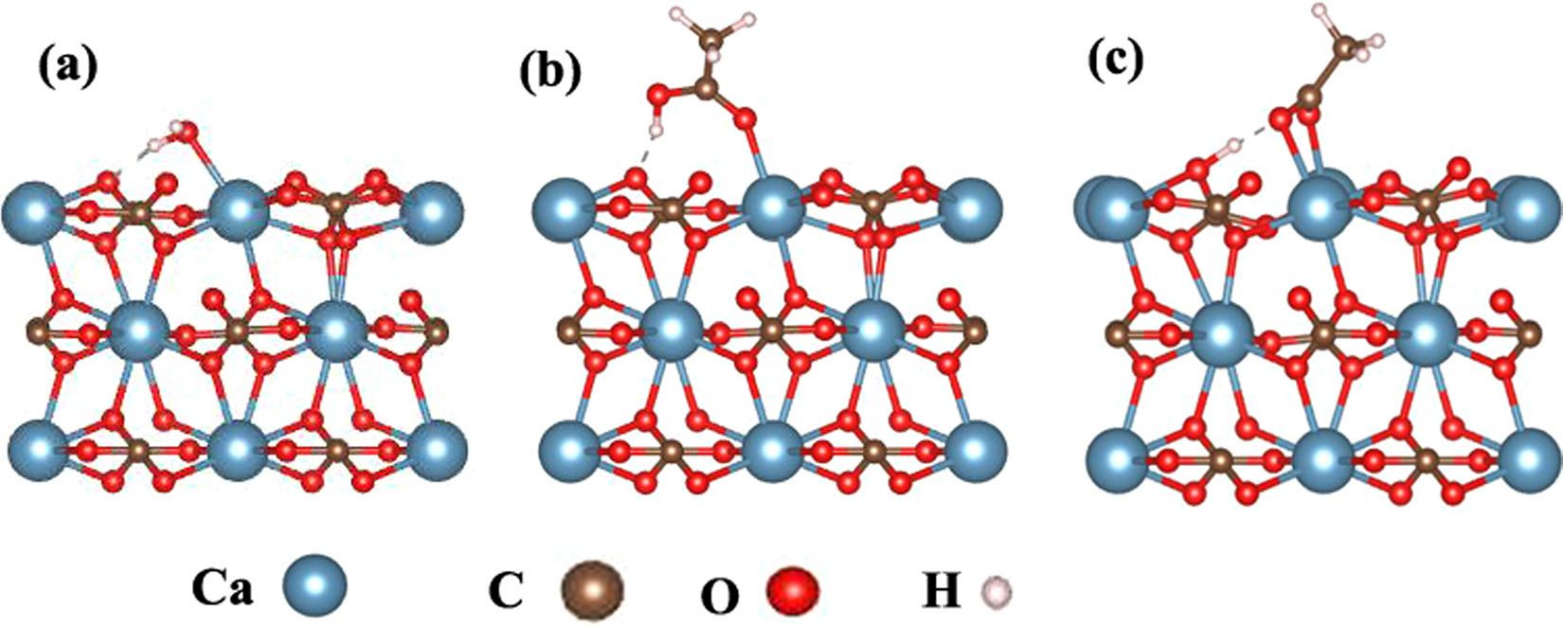
The atomic structures of the adsorption of (**a**) a H_2_O molecule, (**b**) a dissociated acetic acid molecule, and (**c**) an acetic acid molecule.

Oil recovery efficiency is determined by the competition between stickiness (or, the binding energies) of oil and water on the calcite surface in oil wells. Whether a calcite surface is oil-wet or water-wet is determined by the relative binding energy (binding energy difference) between water and oil molecules. For example, if the relative binding energy is −ve (binding energy of oil molecule is larger than water molecule), then one can predicts more oil wet calcite surface. Decrease in relative binding energy due to low salinity water or additive ions of sea water turns calcite surface less oil wet that ultimately enhance crude oil recovery. We first calculate the binding energy of a water and an acetic acid molecules on a calcite surface. Figure 1(a,b) show the optimized water and acetic acid molecules on calcite surface with binding energies of 1.07 eV and 1.28 eV, respectively. These values are in agreement to ref.^4^. The binding mainly arises from the interaction between the O atom in water (acetic acid) and a Ca atom in calcite. There is also weak hydrogen bonding shown by dashed line in Fig. 1. The optimized structure of the dissociative adsorption of H^+^ ion on a calcite surface is shown in Fig. 1(c). The two O atoms in an oil molecule (acetic acid) bind to two Ca atoms in a bidentate configuration. The non-dissociated acetic acid (acetic acid retains its hydrogen (Fig. 1(b)) is stable by 0.08 eV, compared to dissociated acetic acid (acetic acid loses its hydrogen to the calcite surface (Fig. 1(c)). The binding energy of a dissociated acetic acid, (E_*b*_(RCOO^−^)) is 2.10 eV, which suggest that the oil molecule is extracted in the form of non-dissociated acetic acid. Note that the binding of acetic acid is larger than water by 0.21 eV, which confirms that the calcite surface is oil wet (hydrophobic).

**Figure 2:**
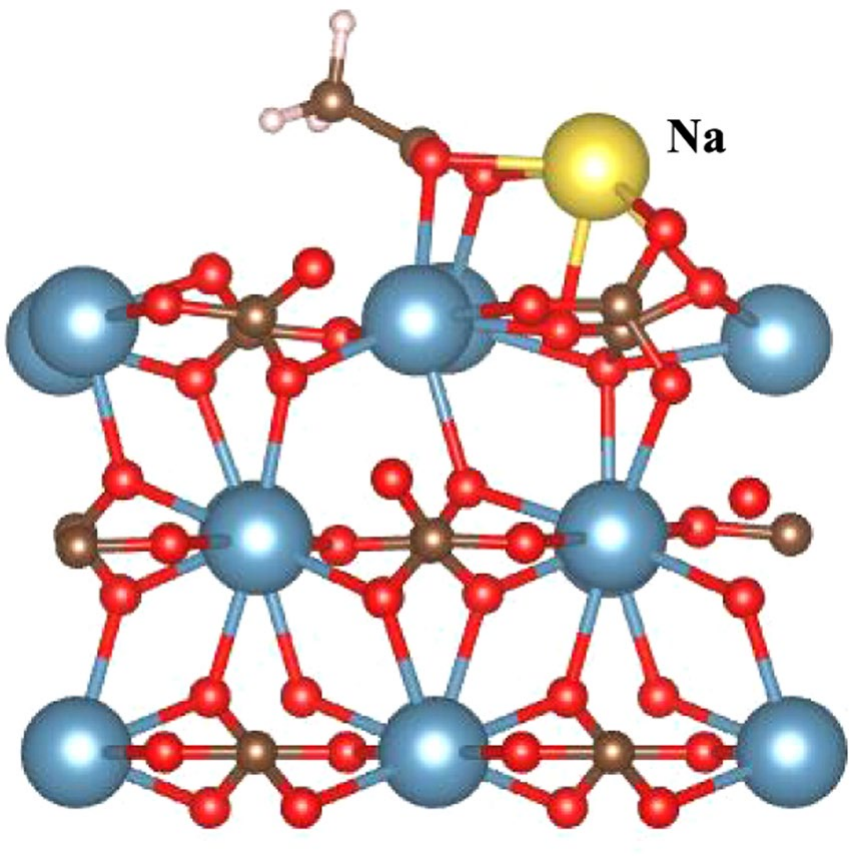
The atomic structure of Na acetate on a calcite surface. The binding energy of Na acetate is 2.04 eV, which is larger than original oil molecule (acetic acid) (2.10 eV vs 1.28 eV). Hence, Na^+^ ions of sea water makes oil molecule more sticker that reduce the crude oil recovery.

Fathi *et al*. in ref.^3^ reported that injecting low salinity sea water (removing some of NaCl from sea water) in oil wells enhances the oil recovery. We consider that Na^+^ can reach to the calcite surface with a pre-adsorbed dissociated acetic acid and forms a Na acetate molecule. The optimized structure of Na acetate on the calcite surface is shown in Fig. 2. The binding energy of Na acetate on a calcite surface is 2.04 eV. Considering that it takes only 1.28 eV to desorb an oil molecule, the existence of Na^+^ makes oil significantly stickier. This provides an explanation to the experimental results in that injecting low salinity sea water (or, removing some of NaCl from sea water) enhances oil recovery^3^.

**Figure 3:**
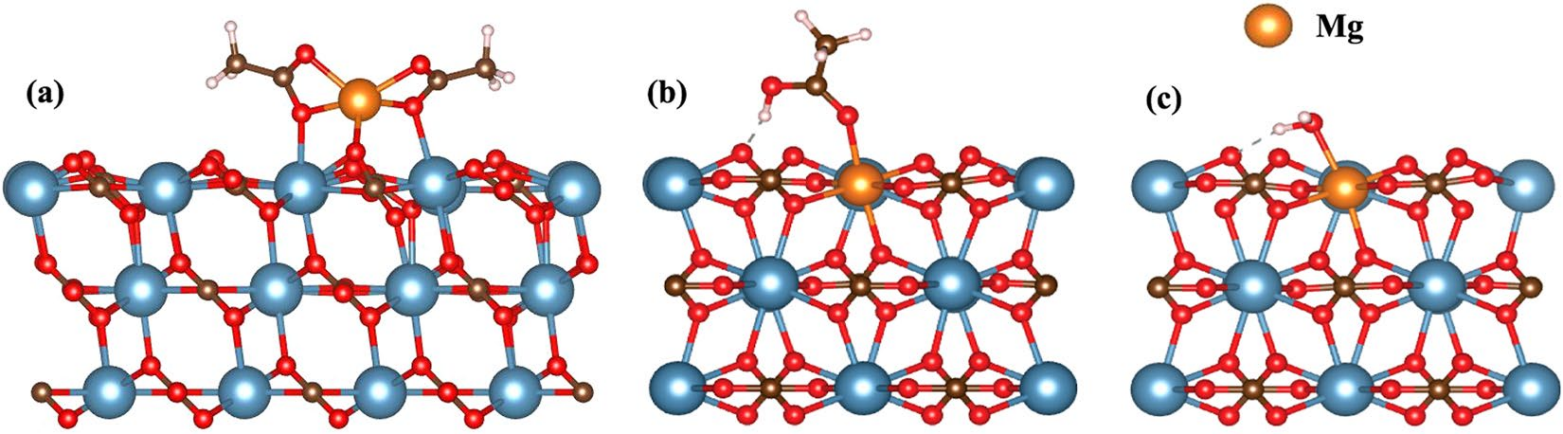
The possible roles of Mg^2+^ ions on oil recovery. The atomic structure of Mg acetate on calcite surface (**a**), acetic acid on modified Mg-calcite surface (**b**) and water molecule on modified Mg-calcite surface (**c**).

In ref.^2^ of Fig. 5, Zhang *et al*. reported that injecting Mg^2+^ alone enhances oil recovery by approximately up to 20%. Note that in ref.^2^ of Fig. 5 on day 53, the temperature was kept constant, which suggest that the enhancement of crude oil recovery is not due to fluid expansion but due to the influence of Mg^2+^ alone. Hence, we investigated the role of Mg^2+^ ions in oil recovery. There are two possibilities for Mg^2+^ ions in oil recovery: (i) Mg^2+^ can reach to the calcite surface near the oil molecule and form Mg-acetate, as shown in Fig. 3(a) and (ii) Mg^2+^ replaces Ca^2+^ as shown in Fig. 3(b) (proposed in refs^2,4^). The binding energy of a Mg acetate molecule is 2.39 eV. As a Mg acetate contains two oil molecules, the effective binding energy of a Mg-modified oil molecule is 1.19 eV. Comparing with the binding energy of the original oil molecule (1.28 eV), oil molecule in the form of Mg-acetate is less sticky which benefits the oil recovery. As suggested in refs^2,4^, Mg^2+^ of sea water may replaces Ca^2+^ of calcite. In this case as shown in Fig. 3(b), the binding energy of acetic acid on modified calcite surface is 1.33 eV but at the same time, the binding energy of water molecule also increases to 1.15 eV, which is shown in Fig. 3(c). Hence, the relative binding energy (binding energy difference between water and oil molecule) on modified Mg-calcite surface is larger than on pure calcite surface (−0.18 eV vs −0.21 eV), which is also beneficial for enhancing oil recovery. However the relative binding energy of Mg-acetate on pure calcite surface is larger than the relative binding energy of acetic acid on modified Mg-calcite surface (−0.12 eV vs −0.18 eV). Hence extracting oil molecule in the form of Mg acetate predicts the experimental result for the enhancement of crude oil by 20% due to having Mg^2+^ alone in oil wells (see ref.^2^ of Fig. 5).

**Figure 4:**
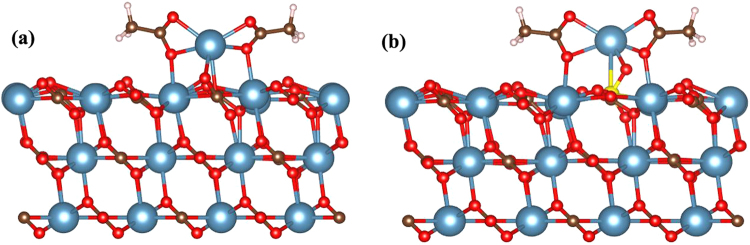
The atomic structures of Ca acetate on pure calcite (**a**) and modified calcite.SO_4_ surfaces (**b**).

**Figure 5:**
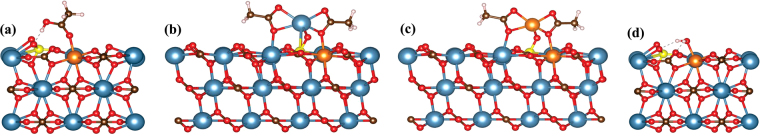
The atomic structures of acetic acid, Ca acetate, Mg acetate and water molecule on modified calcite.MgSO_4_ surface.

In Fig. 4(a), we consider that either replaced Ca^2+^ ions by Mg^2+^ ions during surface modification or excess Ca^2+^ ions of sea water can also reach to the calcite surface and forms Ca acetate. The binding energy of a Ca acetate molecule is 2.52 eV. As a Ca acetate contains two oil molecules, the effective binding energy is 1.26 eV per modified oil molecule. Comparing to the 1.28 eV binding energy of the original oil molecule, the existence of Ca^2+^ ions in seawater make oil less sticky. However, as the energy difference is negligibly small, the effect in oil recovery may not be useful. In ref.^2^ of Fig. 5, Zhang *et al*. also reported that injecting Ca^2+^ along with sulfate ions on day 43 enhances oil recovery by approximately up to 24%. Note that sulfate ions alone have no effect in the oil recovery but oil recovery enhances when sulfate ions along with Ca^2+^ ions injected. Hence, we investigated the role of Ca^2+^ along with sulfate ions in oil recovery. Similar to Fig. 4(a), we consider that Ca^2+^ ions reach to the calcite surface and forms Ca acetate and then ${\rm{S}}{{\rm{O}}}_{4}^{2-}$ ion replaces ${\rm{C}}{{\rm{O}}}_{3}^{2-}$. The optimized structure of Ca acetate on modified calcite.SO_4_ surface is shown in Fig. 4(b). The binding energy of Ca acetate on modified calcite.SO_4_ surface is 2.45 eV and its effective value per modified oil molecule is 1.22 eV. Since the binding energy of original oil molecule is larger than Ca modified oil molecule (1.28 eV vs 1.22 eV), the crude oil recovery is expected to be enhanced.

Finally, we explore the interplay effect of Mg^2+^, Ca^2+^ and ${\rm{S}}{{\rm{O}}}_{4}^{2-}$ ions on oil recovery. Experimental observations in ref.^2^ of Fig. 5 by Zhang *et al*. reported that injecting Mg^2+^ along with ${\rm{S}}{{\rm{O}}}_{4}^{2-}$ ions leads the enhancement of crude oil recovery by approximately up to 32%. Hence, there are three possibilities for the role of Mg^2+^ and ${\rm{S}}{{\rm{O}}}_{4}^{2-}$ ions in oil recovery: (i) Mg^2+^ replaces Ca^2+^ and ${\rm{S}}{{\rm{O}}}_{4}^{2-}$ replaces ${\rm{C}}{{\rm{O}}}_{3}^{2-}$ ref.^2,4^, (ii) the replaced Ca^2+^ ions from calcite and additional Ca^2+^ ions present in sea water can form Ca acetate on modified calcite.MgSO_4_ surfaces, and (iii) the additional left over Mg^2+^ ions that could not find ${\rm{C}}{{\rm{O}}}_{3}^{2-}$ ions during surface modification forms Mg acetate on modified calcite.MgSO_4_ surfaces. In Fig. 5, we have presented the optimized structure of acetic acid (Fig. 5(a)), Ca acetate (Fig. 5(b)) and Mg acetate (Fig. 5(c)) on modified calcite.MgSO_4_ surface. To compare the binding energy difference between water and oil molecule on modified calcite.MgSO_4_ surface, we present the optimized relaxed structure of water molecule on modified calcite.MgSO_4_ surface in Fig. 5(d) that has binding energy of 1.13 eV. The binding energies of acetic acid, Ca acetate per oil molecule and Mg acetate per oil molecule are 1.22 eV, 1.23 eV and 1.15 eV, respectively. By comparing to the binding energy of original oil molecule on pure calcite surface (1.28 eV), the extracted oil molecules in the form of acetic acid, Ca acetate and Mg acetate from the modified calcite.MgSO_4_ surfaces are all helpful for enhancing crude oil recovery. But, oil extraction in the form of Mg acetate from modified calcite.MgSO_4_ surface maximize the enhancement of crude oil recovery because of its lowest binding energy of 1.15 eV.

**Figure 6:**
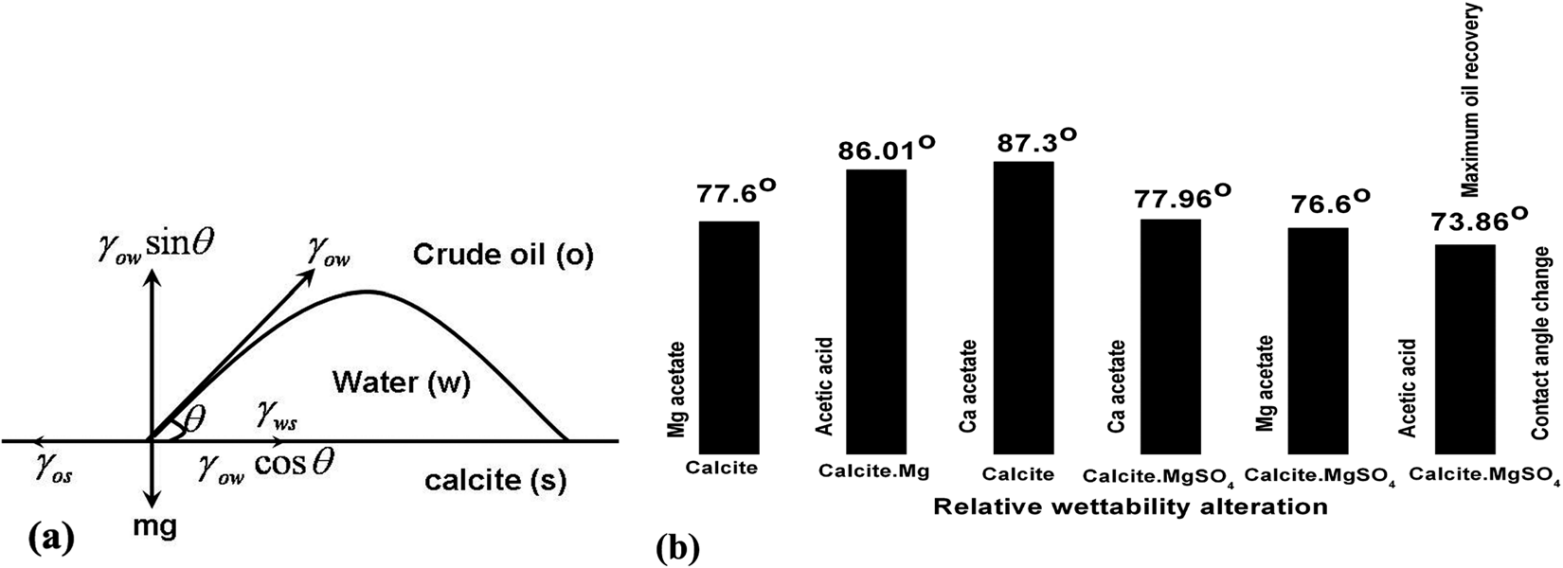
(**a**) Schematic of vectorial picture of a water drop surrounded by crude oil on calcite surface. Since vertically upward acting surface tension force is balanced by vertically acting downward force, we write balanced Young’s equation (2). (**b**) Wettability alteration of calcite surface in terms of contact angle change due to additives ions Mg^2+^, Ca^2+^ and ${\rm{S}}{{\rm{O}}}_{4}^{2-}$ ions of sea water. It can be seen that maximum oil recovery can be achieved for a case when Mg^2+^ replaces Ca^2+^ and ${\rm{S}}{{\rm{O}}}_{4}^{2-}$ replaces ${\rm{S}}{{\rm{O}}}_{4}^{2-}$ that induce hydrophilic (contact angle change ≈ 74°) characteristic of calcite surface. This result completely explain the experimental observations by Zhang *et al*.^2^ of Fig. 5 for the influence of Mg^2+^, Ca^2+^ and ${\rm{S}}{{\rm{O}}}_{4}^{2-}$ ions of sea water in oil recovery.

Enhancement of crude oil recovery is usually determined by measuring contact angle of oil molecule on calcite surface. Contact angles of water droplets on calcite surface in oil wells provides ideal situation to examine the energetics of solid surface. Using Young’s equation^24–26^, from schematic of Fig. 6(a), the contact angle for three phases (calcite surface-oil-water) system can be calculated by the Young’s equation:

2 $${\gamma }_{os}-{\gamma }_{ws}-{\gamma }_{ow}\,\cos \,\theta =\mathrm{0,}$$

where *γ*_*os*_ is the interfacial tension between oil and calcite surface, *γ*_*ws*_ is the interfacial tension between water and calcite surface, *γ*_*ow*_ is the interfacial tension between oil and water. For acetic acid as a model compound of oil molecule, *γ*_*ow*_ = 0.001 *eV*/Å^2^ is used. The change in interfacial tension, Δ*γ* caused by additives Mg^2+^ and ${\rm{S}}{{\rm{O}}}_{4}^{2-}$ ions substitution in proximity, is estimated by the changes in the binding energy (for numerical values, see Table 1) per unit area for oil and water molecule on pure calcite and on modified calcite surfaces as:

3 $${\rm{\Delta }}(\cos \,\theta )=\frac{{\rm{\Delta }}{E}_{os}-{\rm{\Delta }}{E}_{ws}}{{\gamma }_{ow}A},$$

where *A* is the surface area. Since, additive ions Mg^2+^ and ${\rm{S}}{{\rm{O}}}_{4}^{2-}$ present parts per million in sea water, we made approximation that only 5% additive ions participate in the wettability alteration of calcite surface. In Fig. 6(b), the contact angle change on modified calcite surface due to additives Mg^2+^ and ${\rm{S}}{{\rm{O}}}_{4}^{2-}$ ions of sea water from the reference value of the contact angle, *θ* = 90° (mixed oil-water-wet) is shown. In Fig. 6(b), it can be seen that maximum oil recovery can be achieved for a case when Mg^2+^ replaces Ca^2+^ and ${\rm{S}}{{\rm{O}}}_{4}^{2-}$ replaces ${\rm{C}}{{\rm{O}}}_{3}^{2-}$ (contact angle change ≈ 74°). This result explains the experimental observations by Zhang *et al*.^2^ of Fig. 5 in that the interplay between Mg^2+^ and ${\rm{S}}{{\rm{O}}}_{4}^{2-}$ ions in wettability alteration of calcite enhances crude oil recovery. The wettability alteration depends on the presence of Mg^2+^ and ${\rm{S}}{{\rm{O}}}_{4}^{2-}$ ions in sea water. For example, in Table 2, we summarize the contact angle change by considering 1% and 10% coverage of these additive ions in oil wells. Here, it is clear that the contact angle changes from hydrophobic (oil wet) to mixed oil wet (*θ* < 135°) calcite surface.

**Table 1:** Binding energies (eV) of water, acetic acid, Mg acetate and Ca acetate on pure calcite and modified calcite surfaces.

Surface	E_*b*_ (HO)	E_*b*_ (acetic acid)	E_*b*_ (Mg acetate)	E_*b*_ (Ca acetate)
calcite	1.07	1.28	2.39	2.52
calcite.Mg	1.15	1.33	2.38	2.57
calcite.SO_4_	0.96	1.09	2.30	2.45
calcite.MgSO_4_	1.13	1.22	2.31	2.47

The relative binding energy (i.e., the binding energy difference between water and oil molecule) is used in Eq. (3) to calculate the contact angle.

**Table 2:** Change in contact angle due to additive ions of sea water in oil wells for idealistic case of 1% and 10% of wettability alteration.

Oil molecule on surface	contact angle before substitution	*θ* for 1% coverage	*θ* for 10% coverage)
Mg acetate on calcite	90° (mixed wet)	87.6° (mixed wet)	65.4° (water wet)
Mg acetate on calcite	180° (oil-wet)	163.3° (oil wet)	125.7 (mixed wet)
Acetic acid on calcite.Mg	90° (mixed wet)	89.2° (mixed wet)	82.0° (mixed wet)
Acetic acid on calcite.Mg	180° (oil-wet)	170.4° (oil wet)	149.4° (oil wet)
Ca acetate on calcite	90° (mixed wet)	89.4° (mixed wet)	84.7° (mixed wet)
Ca acetate on calcite	180° (oil-wet)	172.2° (oil wet)	155.1° (oil wet)
Ca acetate on calcite.MgSO_4_	90° (mixed wet)	87.6° (mixed wet)	65.3° (water wet)
Ca acetate on calcite.MgSO_4_	180° (oil wet)	163.4° (oil wet)	125.7° (mixed wet)
Mg acetate on calcite.MgSO_4_	90° (mixed-wet)	87.3° (mixed wet)	62.4° (water wet)
Mg acetate on calcite.MgSO_4_	180° (oil wet)	162.5° (oil wet)	122.5° (mixed wet)
Acetic acid on calcite.MgSO_4_	90° (mixed-wet)	86.8° (mixed wet)	56.2° (water wet)
Acetic acid on calcite.MgSO_4_	180° (oil-wet)	160.8° (oil wet)	116.4° (mixed wet)

For idealistic case of solid-liguid-vapor(air) phase, we can express Eq. (2) in terms of work of adhesion,

4 $${W}_{SL}={\gamma }_{LV}(1+\,\cos \,\theta ),$$

where *W*_*SL*_ = *γ*_*SV*_ + *γ*_*LV*_ − *γ*_*SL*_. Here S, V, L corresponds to solid, vapor and liquid phases. By writing work of adhesion in terms of binding energy per unit area, we cal express Eq. 4 as

5 $$1+\,\cos \,\theta =\frac{{E}_{b}}{A{\gamma }_{LV}}\mathrm{.}$$

The contact angle change for water and acetic acid oil molecule is listed in Table 3. Here we have used *γ*_*LV*_ = 72.8 *dyn*/*cm* for water vapor phase and *γ*_*LV*_ = 27.3 *dyn*/*cm* for acetic acid vapor phase. Here we have applied a concept that the larger binding energy on modified calcite surface for water turns the surface towards hydrophilic, where as the larger binding energy for acetic acid turns the surface towards hydrophobic. From Eq. 5, we have estimated the contact angle change and listed in Table 3. Here we also find that the contact angle change for acetic acid turns towards hydrophilic when wettability alterations occutr due to Mg^2+^ and ${\rm{S}}{{\rm{O}}}_{4}^{2-}$ ions.

**Table 3:** Change in contact angle for Solid-Liquid-Vapor (SLV) phases by considering 10% of wettability alteration due to additive ions of sea water and ideal case for initial mixed oil wet calcite surface, *θ* = 90°.

Surface	Contact angle for water	contact angle for acetic acid
Calcite.Mg	84.9° (water wet)	98.4° (oil wet)
Calcite.MgSO_4_	86.2° (less water wet)	79.9° (water wet)

A comment on the molecule extracted in the form of Mg and Ca acetates. In aqueous conditions, these acetate molecules dissociate into acetate ions. Then Mg^2+^ and Ca^2+^ ions can again reach to the oil molecule near the calcite surface and again modify oil molecules in the form of Mg and Ca acetate, which process is acting as a catalyst. Dissociation of Mg and Ca from Mg-acetate and Ca-acetate in water at different temperatures are reported by a number of authors in refs^27,28^.

We have so far investigated the wettability alteration of calcite surface due to additive ions of sea water that has greater influence in the enhancement of crude oil recovery. Now, using quantum molecular dynamics simulations, we investigate the probability of forming the oil molecule as Na, Mg, Ca acetates on the pure calcite and modified calcite.Mg, calcite.SO_4_, calcite.MgSO_4_ surfaces. We use the following reaction mechanism:

6 $$calcite\mathrm{.}RCOOH+N{a}^{+}{({H}_{2}O)}_{n}\rightleftharpoons calcite\mathrm{.}RCOONa+{H}^{+}{({H}_{2}O)}_{n},$$

7 $$calcite\mathrm{.(}RCOOH{)}_{2}+M{g}^{2+}{({H}_{2}O)}_{n}\rightleftharpoons calcite\mathrm{.}Mg{(RCOO)}_{2}+{H}^{+}{({H}_{2}O)}_{n}{H}^{+},$$

8 $$calcite\mathrm{.}RCOOH+M{g}^{2+}{({H}_{2}O)}_{n}\rightleftharpoons calcite\mathrm{.}MgRCOOH+C{a}^{2+}{({H}_{2}O)}_{n},$$

9 $$calcite\mathrm{.(}RCOOH{)}_{2}+C{a}^{2+}{({H}_{2}O)}_{n}\rightleftharpoons calcite\mathrm{.}Ca{(RCOO)}_{2}+{H}^{+}{({H}_{2}O)}_{n}{H}^{+},$$

10 $$\begin{array}{rcl}calcite\mathrm{.(}RCOOH{)}_{2}+S{O}_{4}^{2-}+C{a}^{2+}{({H}_{2}O)}_{n} & \rightleftharpoons  & calcite\mathrm{.}S{O}_{4}Ca{(RCOO)}_{2}\\  &  & +C{O}_{3}^{2-}+{H}^{+}{({H}_{2}O)}_{n}{H}^{+}\mathrm{.}\end{array}$$

11 $$\begin{array}{rcl}calcite\mathrm{.}RCOOH+M{g}^{2+}{({H}_{2}O)}_{n}+S{O}_{4}^{2-}+ & \rightleftharpoons  & calcite\mathrm{.}Mg\mathrm{.}S{O}_{4}RCOOH\\  &  & +C{O}_{3}^{2-}+C{a}^{2+}{({H}_{2}O)}_{n},\end{array}$$

12 $$\begin{array}{l}calcite\mathrm{.(}RCOOH{)}_{2}+C{a}^{2+}{({H}_{2}O)}_{n}+M{g}^{2+}{({H}_{2}O)}_{n}+S{O}_{4}^{2-}\\ \begin{array}{rcl} & \rightleftharpoons  & calcite\mathrm{.}Mg\mathrm{.}S{O}_{4}Ca{(RCOO)}_{2}+C{O}_{3}^{2-}+{H}^{+}{({H}_{2}O)}_{n}{H}^{+}+C{a}^{2+}{({H}_{2}O)}_{n},\end{array}\end{array}$$

13 $$\begin{array}{l}calcite\mathrm{.(}RCOOH{)}_{2}+2M{g}^{2+}{({H}_{2}O)}_{n}+S{O}_{4}^{2-}+\\ \begin{array}{rcl} & \rightleftharpoons  & calcite\mathrm{.}Mg\mathrm{.}S{O}_{4}Mg{(RCOO)}_{2}+C{O}_{3}^{2-}+{H}^{+}{({H}_{2}O)}_{n}{H}^{+}+C{a}^{2+}{({H}_{2}O)}_{n}\mathrm{.}\end{array}\end{array}$$

**Figure 7:**
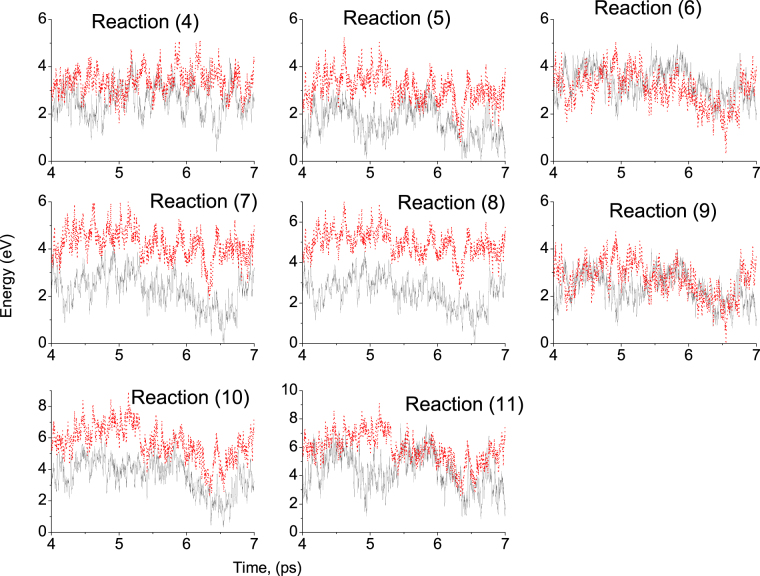
Total energy vs time of reactions (6) to (13). The solid lines (black) are the total energy of the left hand side of reactions (6) to (13) and the dotted lines (red) are the total energy of the right hand side of reactions (6) to (13). We chose zero energy as arbitrary.

In Fig. 7, total energy vs simulation time of left hand side of reactions [(7)–(13)] are shown by solid black lines and right hand side of reactions [(7)–(13)] are shown by red dotted lines. For charged systems, we used a compensating jellium background; which is widely used for total energy calculations of charged systems.

From Fig. 7, it is clear that the reactions (8), (9), (10), (12) are endothermic. It means these reactions do not take place in normal reservoir conditions and thus the additive ions of sea water in the form of calcium acetate and magnesium acetate on pure calcite surface, calcium acetate on calcite.SO_4_ and calcite.MgSO_4_ do not take place. This suggest that the products formed in these reactions may not be energetically preferred and may not have much influence in oil recovery.

On the other hand, from Fig. 7, it is clear that the reactions (6) may be exothermic, where good amount of Na^+^ can reach to the calcite surface and form Na acetate. Since the binding energy of Na acetate is larger than original oil molecule, having Na^+^ ions in sea water clearly reduce the oil recovery. Similarly, from Fig. 7, it is also clear that the reactions (7), (8), (13) may also be exothermic. Hence Mg^2+^ and ${\rm{S}}{{\rm{O}}}_{4}^{2-}$ ions of sea water modify the calcite surface, where the binding energy of oil molecule on these modified calcite srface is smaller than on for pure calcite surface, which enhance the crude oil recovery.

## Conclusions

In summary, using quantum mechanical calculations based on density functional theory, we investigated the role of Na^+^, Ca^2+^, Mg^2+^ and ${\rm{S}}{{\rm{O}}}_{4}^{2-}$ ions of sea water on oil recovery at the atomic scale. We find that Na^+^ ions form Na acetate with oil molecules that make calcite surface more oil-wet which, is harmful to oil recovery. On the other hand, Mg^2+^ and ${\rm{S}}{{\rm{O}}}_{4}^{2-}$ ions of sea water modifies the calcite surface and the additional left over Mg^2+^ ions that could not find ${\rm{S}}{{\rm{O}}}_{4}^{2-}$ ions during surface modification, form Mg acetate on modified calcite.MgSO4 surfaces. This makes the original oil molecule much less sticky on the modified calcite surface. This enhance the crude oil recovery. Finally, by using Quantum Molecular Dynamics simulations, we have shown that having Na^+^ ion in sea water may reach to the calcite surface and form Na acetate (exothermic). We have also shown that surface modification occurs due to having Mg^2+^ and ${\rm{S}}{{\rm{O}}}_{4}^{2-}$ in sea water (exothermic).

## References

[1] Tzimas, E., Georgakaki, A., Cortes, C. G. & Peteves, S. Enhanced oil recovery using carbon dioxide in the european energy system. *Rep*. *EUR* **21895** (2005).

[2] Zhang P, Tweheyo MT, Austad T (2007). Wettability alteration and improved oil recovery by spontaneous imbibition of seawater into chalk: Impact of the potential determining ions ca2+, mg2+, and so42−. Colloids Surfaces A.

[3] Fathi SJ, Austad T, Strand S (2011). Water-based enhanced oil recovery (eor) by ?smart water?: Optimal ionic composition for eor in carbonates. Energy & fuels.

[4] Sakuma H, Andersson MP, Bechgaard K, Stipp SLS (2014). Surface tension alteration on calcite, induced by ion substitution. The J. Phys. Chem. C.

[5] Mohammed M, Babadagli T (2015). Wettability alteration: A comprehensive review of materials/methods and testing the selected ones on heavy-oil containing oil-wet systems. Adv. colloid interface science.

[6] Standnes DC, Austad T (2000). Wettability alteration in chalk: 2. mechanism for wettability alteration from oil-wet to water-wet using surfactants. J. Petroleum Sci. Eng..

[7] He L, Lin F, Li X, Sui H, Xu Z (2015). Interfacial sciences in unconventional petroleum production: from fundamentals to applications. Chem. Soc. Rev..

[8] Winsor, P. A. *Solvent properties of amphiphilic compounds* (Butterworths Scientific Publications, 1954).

[9] Ehrlich R, Wygal RJ (1977). Interrelation of crude oil and rock properties with the recovery of oil by caustic waterflooding. Soc. Petroleum Eng. J..

[10] Gupta R, Mohanty K (2010). Temperature effects on surfactant-aided imbibition into fractured carbonates. Soc. Petroleum Eng. J..

[11] Karimi M, Mahmoodi M, Niazi A, Al-Wahaibi Y, Ayatollahi S (2012). Investigating wettability alteration during meor process, a micro/macro scale analysis. Colloids Surfaces B: Biointerfaces.

[12] Nasralla RA, Bataweel MA, Nasr-El-Din HA (2013). Investigation of wettability alteration and oil-recovery improvement by low-salinity water in sandstone rock. J. Can. Petroleum Technol..

[13] RezaeiDoust A, Puntervold T, Strand S, Austad T (2009). Smart water as wettability modifier in carbonate and sandstone: A discussion of similarities/differences in the chemical mechanisms. Energy & fuels.

[14] Lardge JS, Duffy D, Gillan M (2009). Investigation of the interaction of water with the calcite (10.4) surface using ab initio simulation. The J. Phys. Chem. C.

[15] Lardge JS, Duffy DM, Gillan MJ, Watkins M (2010). Ab initio simulations of the interaction between water and defects on the calcite (101? 4) surface. The J. Phys. Chem. C.

[16] Andersson MP, Stipp SLS (2012). How acidic is water on calcite?. The J. Phys. Chem. C.

[17] Andersson MP, Sakuma H, Stipp SLS (2014). Strontium, nickel, cadmium, and lead substitution into calcite, studied by density functional theory. Langmuir.

[18] Andersson, M. P., Dideriksen, K., Sakuma, H. & Stipp, S. L. S. Modelling how incorporation of divalent cations affects calcite wettability–implications for biomineralisation and oil recovery. *Sci*. *Reports* **6** (2016). 10.1038/srep28854 PMC4926276 27352933

[19] Giannozzi P (2009). Quantum espresso: a modular and open-source software project for quantum simulations of materials. J. Physics: Condens. Matter.

[20] Perdew JP, Burke K, Ernzerhof M (1996). Generalized gradient approximation made simple. Phys. Rev. Lett..

[21] Grimme S (2006). Semiempirical gga-type density functional constructed with a long-range dispersion correction. J. computational chemistry.

[22] Heberling F (2011). Structure and reactivity of the calcite–water interface. J. colloid interface science.

[23] Momma K, Izumi F (2008). Vesta: a three-dimensional visualization system for electronic and structural analysis. J. Appl. Crystallogr..

[24] Young T (1805). An essay on the cohesion of fluids. Philos. Transactions Royal Soc. Lond..

[25] de Gennes PG (1985). Wetting: statics and dynamics. Rev. Mod. Phys..

[26] Erbil HY (2014). The debate on the dependence of apparent contact angles on drop contact area or three-phase contact line: A review. Surf. Sci. Reports.

[27] Rivett ACD (1926). The constitution of magnesium acetate solutions. J. Chem. Soc. (Resumed).

[28] Pezeshki S, Lin H (2014). Molecular dynamics simulations of ion solvation by flexible-boundary qm/mm: On-the-fly partial charge transfer between qm and mm subsystems. J. computational chemistry.

